# Accelerometers in Our Pocket: Does Smartphone Accelerometer Technology Provide Accurate Data?

**DOI:** 10.3390/s23010192

**Published:** 2022-12-24

**Authors:** George Grouios, Efthymios Ziagkas, Andreas Loukovitis, Konstantinos Chatzinikolaou, Eirini Koidou

**Affiliations:** 1 Department of Physical Education and Sport Science, Aristotle University of Thessaloniki, 57001 Thessaloniki, Greece; 2 Department of Physical Education and Sport Science-Serres, Aristotle University of Thessaloniki, Agios Ioannis, 62110 Serres, Greece

**Keywords:** smartphones, performance evaluation, accelerometer sensors, accelerometer accuracy, optoelectronic system, gait

## Abstract

This study evaluates accelerometer performance of three new state of the art smartphones and focuses on accuracy. The motivating research question was whether accelerator accuracy obtained with these off-the-shelf modern smartphone accelerometers was or was not statistically different from that of a gold-standard reference system. We predicted that the accuracy of the three modern smartphone accelerometers in human movement data acquisition do not differ from that of the Vicon MX motion capture system. To test this prediction, we investigated the comparative performance of three different commercially available current generation smartphone accelerometers among themselves and to a gold-standard Vicon MX motion capture system. A single subject design was implemented for this study. Pearson’s correlation coefficients^®^ were calculated to verify the validity of the smartphones’ accelerometer data against that of the Vicon MX motion capture system. The Intraclass Correlation Coefficient (ICC) was used to assess the smartphones’ accelerometer performance reliability compared to that of the Vicon MX motion capture system. Results demonstrated that (a) the tested smartphone accelerometers are valid and reliable devices for estimating accelerations and (b) there were not significant differences among the three current generation smartphones and the Vicon MX motion capture system’s mean acceleration data. This evidence indicates how well recent generation smartphone accelerometer sensors are capable of measuring human body motion. This study, which bridges a significant information gap between the accuracy of accelerometers measured close to production and their accuracy in actual smartphone research, should be interpreted within the confines of its scope, limitations and strengths. Further research is warranted to validate our arguments, suggestions, and results, since this is the first study on this topic.

## 1. Introduction

Smartphones are a new class of mobile (portable) or other cellular multimedia devices that provide integrated services from modern communication, computing, information, and mobile technology into one unit. Smartphones are increasingly intertwined into the fabric of modern society in all spheres of human private and public life, including science, technology, health, psychology, environment, economy, education, culture and art.

Life in the contemporary world is becoming more and more digital, networked, and smartphone centric. As such, the world is literally at humans’ fingertips. Our lives are controlled with light finger taps on tiny smartphone screens; hence, smartphones are rarely far from hand. There is no doubt that the scope of the smartphone utility has expanded beyond what was originally anticipated. The paper-thin smartphones of today are used as multipurpose devices offering every feature users desire worldwide. It is not surprising, therefore, that the smartphone is described the as the “Swiss army knife for the 21st century” [1].

Smartphones come equipped with an array of microelectromechanical sensors (MEMS) capable of gathering information about the world around them, such as location-based and motion sensors, environmental sensors, biometric sensors and activity and health sensors. Current generation smartphones incorporates diverse and powerful sensors, which are small in size and low cost, consume little energy, and feature high performance, making them increasingly sophisticated and advanced.

Smartphone sensors are specific technologies able to sense a physical quantity and translate it into electric signals, so that the particular quantity can be interpreted by the smartphone computing system. They are, in effect, self-sensing devices aware of their surrounding environment in real time, largely independent of location and time, operating in an unobtrusive manner for the user [2]. With modern smartphones being open and programmable, software developers have access to these sensors to create novel sensing applications of increasing functionality and complexity in a wide range of fields, including, but not limited to, health [3], rehabilitation [4], physical activity [5], social networks [6], environment [7], transportation [8] and safety [9]; thus, mobile phone sensing became a new field for scientific and clinical research [10].

Smartphone sensors can be categorized into two main types, based on their system design and communication features: internal (built-in or embedded) sensors and external sensors [11]. The internal sensors can be divided into two main groups: raw sensors, which permit capturing data directly from hardware sensors, embedded into the device, and derived sensors which provide processed and fused data from a few raw sensors all at once, providing users of the system with different information [10,12]. Accordingly, the external sensors can be grouped into two major kinds: wire sensors, which are physically connected to a USB port or dock connector, and wireless sensors, which are connected via Bluetooth to a wearable sensing module [11].

Among smartphone sensors, the accelerometer is one of the earliest and most ubiquitous [13]. Recent generation of smartphones include MEMS-based accelerometer sensors by default. The accelerometer sensor measures constant (gravity), time varying (vibrations) and quasi static (tilt) acceleration forces, which affect the device on the three axes (x, y and z) in meter per second squared (m/s^2^) [14].

Currently, with all these characteristics, smartphone accelerometers have been widely used as a useful and powerful tool for laboratory [15] and field [16] research and have also opened up new possibilities for mobile-sensing research [13]. A particular advantage of utilizing modern smartphone accelerometer technology, is its ability, at least under certain circumstances, to enhance testing schemes and influence measurement strategies [17], as well as to replace more expensive scientific instruments and more established inertia equipment [18].

However, performance variations of smartphone accelerometers across, e.g., manufacturers, models, applications, operating system types and central processing unit (CPU) conditions, lead to heterogeneities in the collected data [13]. The heterogeneities in smartphone accelerometer sensing, along with the issues and challenges they come with, have been investigated in previous studies.

Douangphachanh and Oneyama [19], for instance, estimated road roughness condition using data collected by sensors from two different smartphones loosely placed within a moving vehicle at realistic locations and under realistic manner. The results confirmed that road roughness condition was linearly related to acceleration magnitude and average speed. They also revealed that the strength of this relationship varies markedly at different frequency ranges.

Dey et al. [20] hypothesized that due to hardware imperfections during the sensor manufacturing, smartphone and tablet accelerometers possess unique fingerprints which can be exploited for tracking users. To this end, they measured and classified 80 standalone accelerometer chips, 25 android phones, and 2 tablets. The results provided confirmation that such fingerprints exist, and are readily visible even in uncontrolled, real-life settings.

Douangphachanh and Oneyama [21] collected accelerometer and gyroscope data from four common smartphones installed at three different locations within four separate vehicles running on road sections with varying roughness conditions. The magnitudes of vibration, calculated from each axis of the accelerometers and gyroscopes, as well as the average speed, were found to be strongly related with road roughness conditions. Modeling the road roughness condition as a linear function of vibration magnitudes, which includes both accelerometer and gyroscope data as well as average speed, offered better estimation results than models that only take into account accelerometer magnitudes and average speeds.

Feng et al. [22] investigated the use of three mainstream smartphone embedded accelerometers for monitoring structural vibration and diagnosing structural health and post-event damage. In both the temporal and frequency domains, the results demonstrated good agreement between the reference and smartphone sensor readings, indicating the smartphone sensors’ capacity to measure structural responses ranging from low-amplitude ambient vibration to high-amplitude seismic response.

Mourcou et al. [23] evaluated the performance accuracy of three sensors and algorithms embedded in three different smartphones against a very specific benchmark, a true-to-standard industrial robot arm that utilizes inertial motion units for clinical research. These comparisons were made using two protocols: static and dynamic. The findings indicated that the two protocols were not affected by filters and hardware effects. In addition, they revealed that the smartphone performance results were comparable to those of the benchmark.

Stisen et al. [13] examined sensor-, device- and workload-specific heterogeneities, focusing on accelerometer sensors, employing 31 smartphones, one tablet and four smartwatches, depicting 13 different models of four different manufacturers running variants of iOS and Android. Additionally, they carried out tests with nine users and considered popular feature representation and classification techniques in research on recognition of human activity. The results suggested that on-device sensor and sensor handling heterogeneities significantly impair human activity recognition research performances. Furthermore, the impairments differ considerably between devices and are dependent on the type of the recognition technique that is used.

Figueiredo et al. [24] assessed the ability of smartphone built-in sensors to distinguish between fall events and activities of daily living. Specifically, they explored the fall information provided by the accelerometer, magnetometer, and gyroscope sensors in two smartphone models of the same brand. In their research, accelerometer was found to be the most reliable sensor. Using the data provided by this sensor, a novel, simple and reliable fall detection algorithm was proposed using a threshold-based approach. A comparative study carried out on the same dataset with other existing smartphone-based fall detection algorithms showed that the proposed algorithm was very competitive.

Kos, Tomažič, and Umek’s [25] study aimed to address whether smartphone inertial sensor performance varies considerably among different smartphone models. Accordingly, they evaluated the accuracy of 116 different smartphone devices of 61 different models, all from 13 different manufacturers, and figured out biases between measurements from smartphone devices. The results revealed that the measured parameters for smartphone sensors were highly variable between smartphone models and, in some cases, even within the same model.

Chen et al. [26] tested the effect of the specifications of four built-in smartphone accelerometers of different brands and models on fall detection performance. An algorithm for detecting falls was built, and its accuracy was then calculated and compared on these smartphones. The results showed that the fall detection algorithm had varying values of sensitivity and specificity when performed by smartphones of different manufacturers.

Kask and Kuusik [27] compared the performance accuracy of two conventional smartphones and a specialized wearable sensor employed in prior clinical research. The results of two specific clinical tests, the Romberg and the range of motion test, were analyzed. No significant differences were found between the motion range measurements of the used reference sensor and the two used smartphones. It was suggested that both contemporary smartphones are adequate for angular motion detection and can serve as replacements for specialized wearable equipment to assess human body movements.

Ahmed et al. [28] investigated measurement variability among smartphone sensors from three different brands. A field experiment using the same vehicle, device mounting method, traversal speed, and roughness index method demonstrated that accelerometer sensitivities and maximum sample rates vary significantly within and between brands of smartphones. The study found that calibrating smartphones is necessary for accurate roughness measurements to be consistent across different models.

Kuhlmann, Garaizar, and Reips [29], using newly designed measurement equipment, tested the accuracy of orientation data regarding the spatial position of 56 distinct smartphones. In addition, native apps were used to measure a subsample of 39 devices. With the help of a software sensor, location data was gathered by integrating information from different sensors on the smartphone to produce gravity acceleration data. Data gathered from sensors via web browsers and native apps was compared to the objective status of the smartphone’s vertical and horizontal orientation. The results obtained supported the view that data collected from a variety of smartphone devices revealed heterogeneity in orientation information.

Yang’s et al. [30] study aimed to identify differences in the sensitivity of smartphone inertial sensors, which might result in measurement inconsistencies. Thus, they evaluated three calibration methods for calibrating the road roughness measurements using three different types of reference smartphones for both paved and unpaved roads. It was demonstrated that roughness indices from each device and road type were normally distributed with unequal means under identical conditions of device mounting and vehicle use.

Evidently, these existing studies evaluate performance variations of smartphone accelerometers across different manufacturers, models and types. Such evaluations were crucially needed to allow or not the use of the modern smartphone accelerometers to perform experimental and clinical measurements in laboratory and field settings. However, despite the significant research interest that has been devoted to assessing comparative performance of previous generation smartphone accelerometers, there has been no study, to the best of our knowledge, on their comparative or individual performance of recent generation smartphone accelerometers against a gold-standard optoelectronic system, such as the Vicon MX motion capture system (Vicon Motion Systems, Oxford, UK). The purpose of the current study was to address this lack of information by rigorously and comprehensively investigating the comparative performance of three different commercially available modern smartphone accelerometers amongst themselves and to a gold-standard Vicon MX motion capture system. The hypothesis that accelerator accuracy in human movement data acquisition obtained with three new state-of-the-art smartphone accelerometers and the gold-standard Vicon MX motion capture system was not statistically different was tested.

## 2. Materials and Methods

### 2.1. Sample

In order to eliminate or minimize threats to internal validity of the study, a single subject design was implemented for this study. This design allows the researchers to eliminate and hold constant extraneous variables, such as subjects’ sex, height, weight and footwear that might otherwise contaminate the results of the study. The subject was a 29-year-old male Ph.D. research student of mass 72 kg and height 1.78 m. He carefully screened for health status and was found to be in good general health (he did not have a record or symptoms of psychiatric or neurological illness, physical or orthopaedic injury, or specific deficits in motor control).

The subject was naive as to the purpose of the study and agreed to participate voluntarily with written and signed informed consent. He was informed about his right to withdraw at any time for any reasons during the study, without any negative consequences, and was guaranteed privacy and confidentiality of all gathered information and the anonymous presentation of findings. Additionally, he was assured that the study would not have any physical, psychological, social, or ethical risks. The subject was not withdrawn from the study despite withdrawal of consent. Data collection was carried out in a quiet and comfortable motor-behavior laboratory, in a nonstressing condition, under the researchers’ supervision.

### 2.2. Instruments

Three of the most widely used brands and generations of smartphones, referred to as Smartphone 1 (iPhone 12 Pro Max), Smartphone 2 (Samsung Galaxy S21 Ultra) and Smartphone 3 (Huawei P Smart), were tested in this study (Table 1). Accelerometer data was collected using the Apple’s iOS application “Accelerometer” (by DreamArc), installed on Smartphone 1 and the Google’s Android application “Accelerometer Acceleration Log” (by Alfa V), installed on Smartphone 2 and Smartphone 3. Both applications allow users to screen, record and save triaxial acceleration data on a smartphone device and export them to a semicolon separated CSV file (“Accelerometer” application) or to a comma separated CSV file (“Accelerometer Acceleration Log” application).

The sampling frequency of the “Accelerometer” application is adjustable from 1 Hz to 30 Hz and of the “Accelerometer Acceleration Log” from 1 Hz to 60 Hz. The Vicon MX motion capture system with 10 Bonita 3 optoelectronic cameras was used to record three-dimensional data. Known as one of the most advanced digital optical motion capture systems available today [31], this system is widely accepted as the gold standard for gait assessment and multifactorial movement analysis [32]. The Vicon MX motion capture system operates in the near-infrared spectrum and precisely records the 3D positions of reflective markers within a millimeter accuracy and with a measurement update frequency above 100 Hz in a capture volume of 6 × 6 × 2.5 m. The system calculates the accurate 3D locations of sparse reflective markers attached at bony anatomical landmarks of the subject’s body according to the standard Vicon full-body marker placement protocol called Plug-In-Gait (PIG) [33] to recover the body’s original shape and pose. The facility is supplied with Vicon’s Nexus v. 1.8.5 software (Vicon Motion Systems, Oxford, UK) with the Full-Body Plug-in Gait marker placement model, which is used to setup and calibrate the system and capture and process data.

### 2.3. Procedure

**Figure 1:**
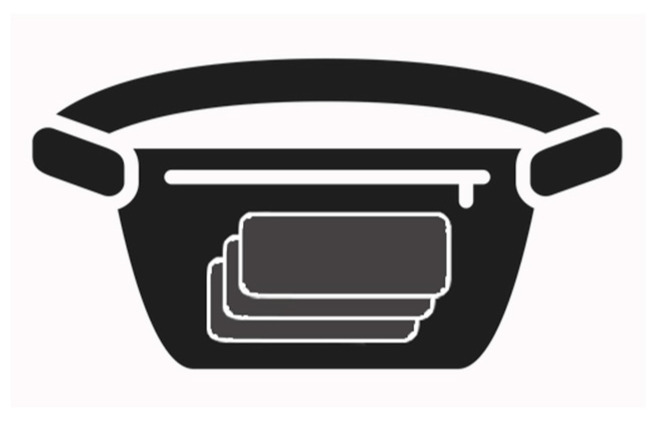
Smartphone rugged belt holster carrying case.

Data collection was conducted in one day, in a 4-h period, at the Laboratory of Motor Behavior and Adapted Physical Activity of the Department of Physical Education and Sport Sciences of the Aristotle University of Thessaloniki, Greece. At first, the three smartphones were placed in a 3-piece (sandwich-style) smartphone horizontal, rugged belt holster carrying case, firmly attached to the lumbar spine (lower back) via an elastic belt (Figure 1).

**Figure 2:**
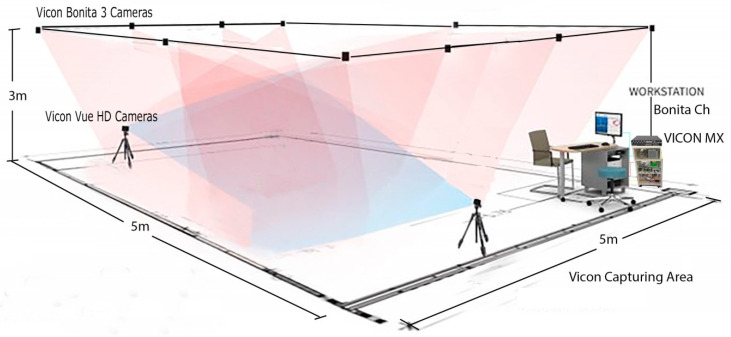
Vicon system capturing area.

To reduce the impact of external influences on accelerometer-sensing heterogeneities, the data for this investigation was collected while maintaining the CPU load on all cellphones to a minimum (i.e., only running the data collection application) [34]. Then, after collecting somatometric data of the subject, including weight, height and various extremity length metrics, we calibrated the Vicon system capturing area (5 m × 5 m × 3 m) (Figure 2) and started capturing.

**Figure 3:**
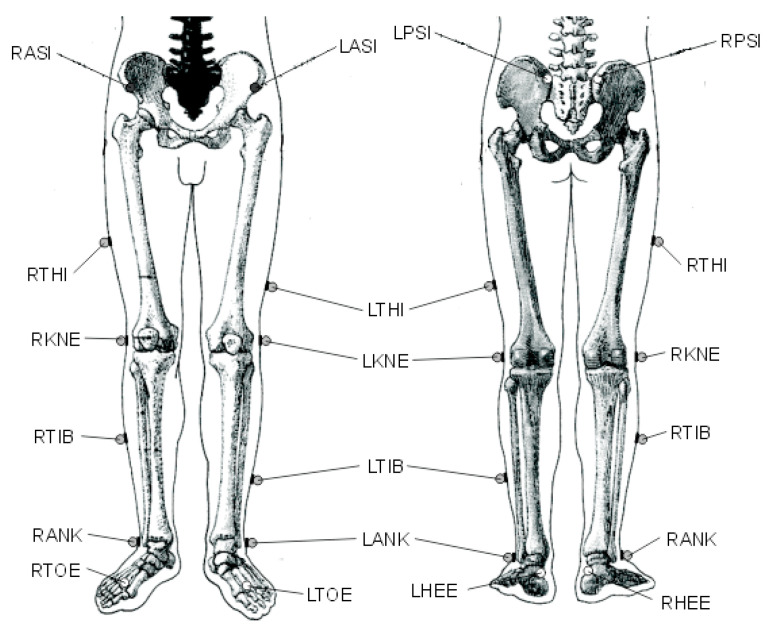
Reflective marker placement according to the Plug-In-Gait lower limbs model.

The capturing procedure lasted approximately 45 min. The capturing frequency of the Vicon MX motion capture system and the three smartphones was set to 15 Hz. For the 3D video capture, we used the PIG (lower limbs model) [33] reflective market placement using a total of 16 reflective markers placed on specific anatomical points of subject’s body (Figure 3).

**Figure 4:**
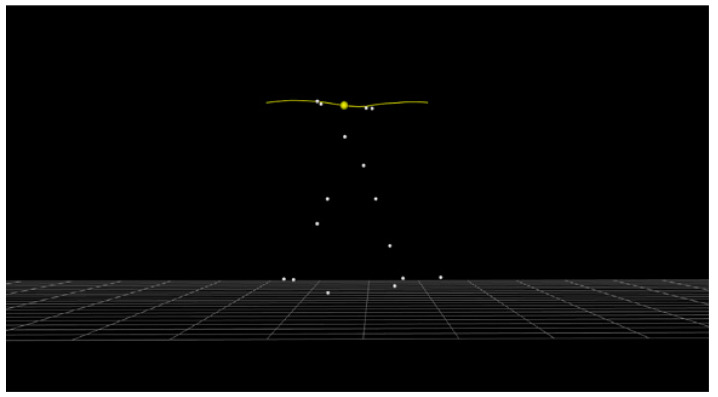
Lateral view of the subject performing the walking task.

Before capturing gait trials, the subject was able to perform some walking trials in order to become acquainted with the space environment and the process. Subsequently, we performed a static subject calibration. Then we captured 9 gait trials (18 steps) from the subject performing a 6 m walkway at his preferred (also known as the “spontaneous” or “self-selected”) walking speed inside the Vicon system capturing area (Figure 4).

**Figure 5:**
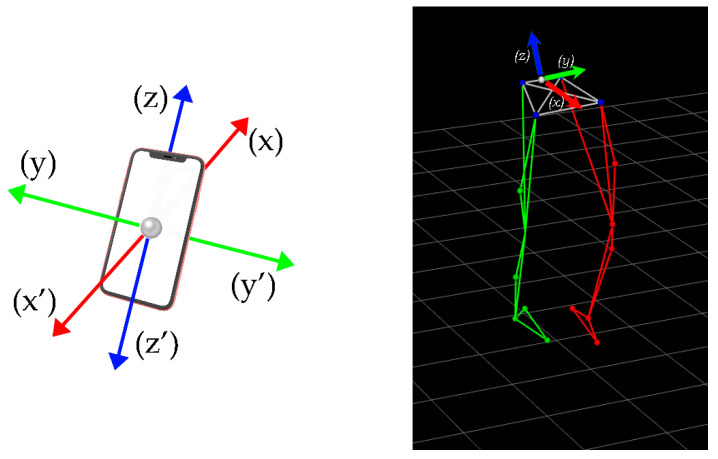
Smartphones and average market orientation set.

The first 3 gait trials (six steps) of the captured walking task were used to verify the validity and reliability of the variables of interest (i.e., the acceleration forces). The position of the 10 infrared cameras remained unchanged from one gait trial to the next. Marker position data along the three axes were assimilated using Vicon system software (Nexus v. 1.8.5, Oxford, UK). Aiming at facilitating the post smartphones’ accelerometers data synchronization, we asked the subject to start and stop each gait trial from the standing position. In order to synchronize accelerometers’ raw data, the sampling rate of all devices used in this experimental design was set to 15 Hz. In addition, the offset of each device in each trial was calculated from the start of each recording till the beginning of gait. Six walking steps were captured for each gait trial. After the subject’s performance of the 9 gait trials, we collected the accelerometers’ data from the three smartphones. Following the 3D video capturing, we labelled reflective markers based on the lower body plug in gait model and exported data concerning trajectories of each reflective marker (Figure 5). After the post capturing data processing, we exported a c3d format type file containing the trajectories of all reflective markers for further analysis.

### 2.4. Data Processing and Statistical Analysis

Accelerometer data from the three smartphones were exported on a CSV file format from each trial. At the end of the performance task, we collected 27 CSV files (9 CSV files from each smartphone). Post captured acceleration data from the Vicon system were calculated. Specifically, the c3d files created by the Vicon system were opened and edited using the Mokka (Motion Kinematic and Kinetic Analyzer, version 0.6.2) software. From the c3d file we created an average marker from the two reflecting markers (LPSI and RPSI), which were placed on the slight bony prominences that can be felt immediately below the dimples (sacroiliac joints), at the point where the spine joins the pelvis. From the trajectories of the average marker on the three-axis system we calculated the lineal acceleration using the following formulas:

(1) $$a\left(x\right)=\frac{d\left(\frac{dx}{dt}\right)}{dt},\;a\left(y\right)=\frac{d\left(\frac{dy}{dt}\right)}{dt},\;a\left(z\right)=\frac{d\left(\frac{dz}{dt}\right)}{dt}$$

All acceleration data were then imported in a data file in the statistical software platform “Statistical Package for the Social Sciences” (SPSS) (26.0). Descriptive statistics were conducted including means and standard deviations of acceleration in each axis of each device and Pearson’s correlation coefficients (r) (i.e., a measure of the strength of a linear association between two continuous variables). Furthermore, the acceleration magnitude (AM) of each device was calculated in a single worksheet in Microsoft excel for each axis using the formula $AM=\sqrt{x^{2}+y^{2}+z^{2}}$, as has already been described in previous research [35,36]. The intraclass correlation coefficient (ICC) was used in order to test the absolute agreement between acceleration data from all devices used in this experiment using the two-way mixed effects model. The appropriate forms of the ICC were the ICC intra-rater reliability, absolute agreement, as reported by Shrout and Fleiss [37]. This ICC illustrated the absolute agreement for multiple measurements and was generally considered as being either poor, moderate, good, or excellent reliability for values less than 0.5, between 0.5 and 0.75, between 0.75 and 0.9, and greater than 0.90, respectively [38]. The *p*-value for both the Pearson’s correlation coefficients (r) and the ICC was set at the level of 0.05. Finally, inferential statistics [namely, a three-way repeated measures ANOVA (9 trials × 4 devices × 3 axis)] was used to test our hypothesis. The threshold for significance was fixed at 0.05.

## 3. Results

### 3.1. Descriptive Statistics

Descriptive statistics concerning each device acceleration data for the three axes during the six steps trial are presented in Table 2.

**Figure 6:**
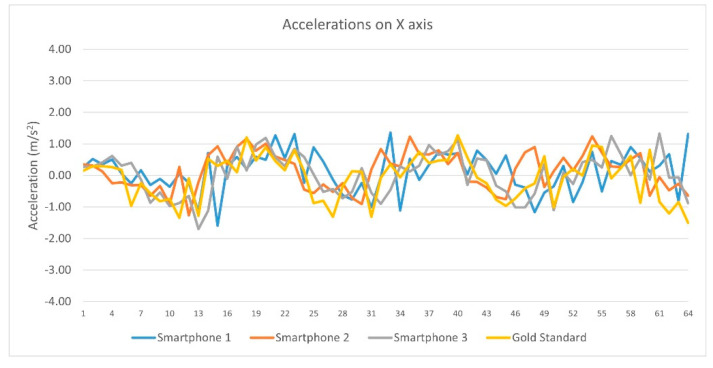
Pearson r correlations from each device linear acceleration’s data on the X axis.

Regarding linear acceleration data from each device on the x axis, Pearson’s correlation coefficient (r) showed significant correlations between (a) Smartphones 1 and Vicon (Pearson r = −0.409, sig = 0.039), (b) Smartphones 2 and Vicon (Pearson r = −266, sig = 0.025), (c) Smartphone 3 and Vicon system (Pearson r = −0.464, sig = 0.000). Pearson r correlation from each device linear acceleration’s data on the X axis are presented in Table 3 and Figure 6.

**Figure 7:**
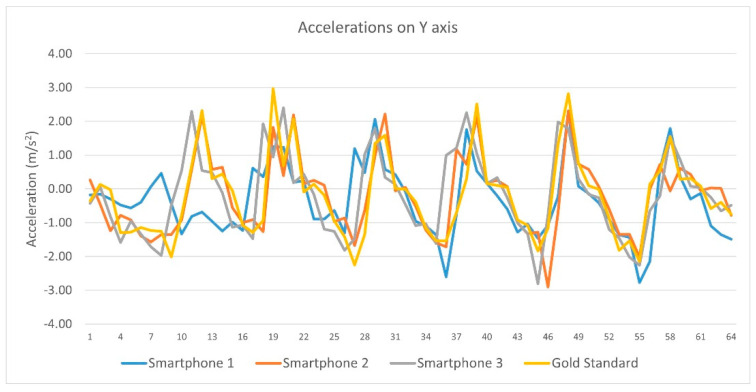
Pearson r correlations from each device linear acceleration’s data on the Y axis.

Concerning linear acceleration data from each device on the Y axis, Pearson’s correlation coefficient (r) showed significant correlations between (a) Smartphone 1 and Vicon system (Pearson r = −0.415, sig = 0.000), (b) Smartphone 2 and Vicon system (Pearson r = −0.354, sig = 0.002) and (c) Smartphones 3 and Vicon system (Pearson r = 0.292, sig = 0.001). Pearson r correlation from each device linear acceleration’s data on the Y axis are displayed in Table 4 and Figure 7.

**Figure 8:**
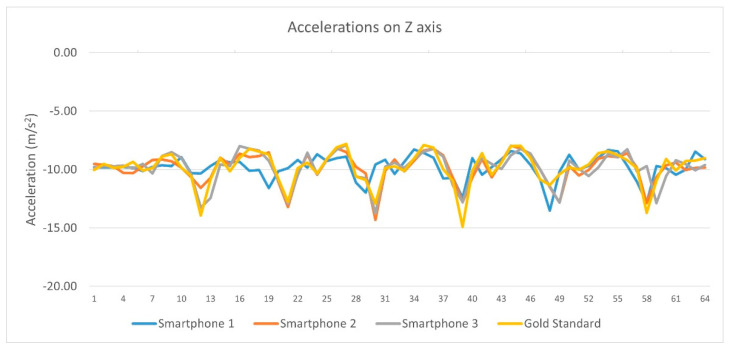
Pearson r correlations from each device linear acceleration’s data on the Z axis.

Referring to linear acceleration data from each device on the Z axis, Pearson’s correlation coefficient (r) showed significant correlations between (a) Smartphone 1 and Vicon system (Pearson r = −0.306, sig = 0.009), (b) Smartphone 2 and Vicon system (Pearson r = −0.255, sig = 0.032) and (c) Smartphone 3 and Vicon system (Pearson r = −0.330, sig = 0.002). Pearson r correlation from each device linear acceleration’s data on the Z axis are depicted in Table 5 and Figure 8.

**Figure 9:**
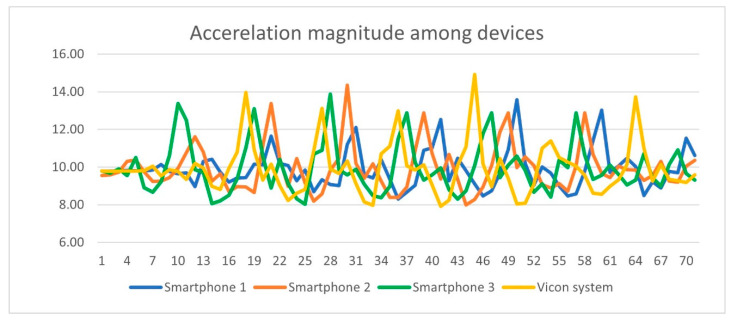
Intraclass Correlation Coefficient values among device’s acceleration magnitude.

Pertaining to the Intraclass Correlation Coefficient (ICC) of the acceleration magnitude among the four devices used in this experiment, ICC values ranged from −0.348 to 0.796. Specifically, acceleration magnitude ICC between (a) Smartphones 1 and Vicon system was −0.348, sig = 0.008 (b) Smartphones2 and Vicon system was 0.796, sig = 0.001, (c) Smartphone 3 and Vicon system was 0.270, sig = 0.001 All ICC measures were statistically significant at *p* < 0.05. ICC values concerning acceleration magnitude of the four devices are illustrated in Table 6 and portrayed in Figure 9.

### 3.2. Inferential Statistics

**Figure 10:**
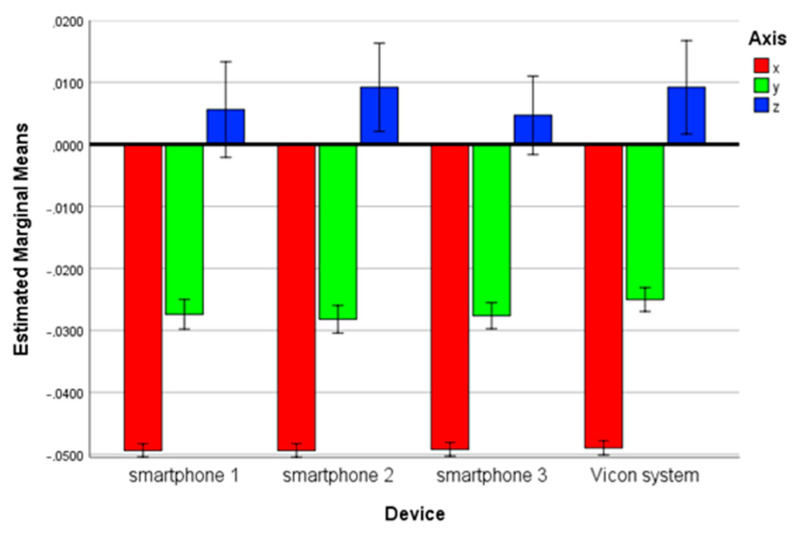
Estimated marginal means of the three-axis acceleration among the three smartphones and the Vicon system.

There was no statistically significant three-way interaction between device, axis and step F_(30,288)_ = 1,302, *p* = 0.140. There was, also, no statistically significant two-way interaction between device and axis F_(6,288)_ = 0.408, *p* = 0.874 (Figure 10).

**Figure 11:**
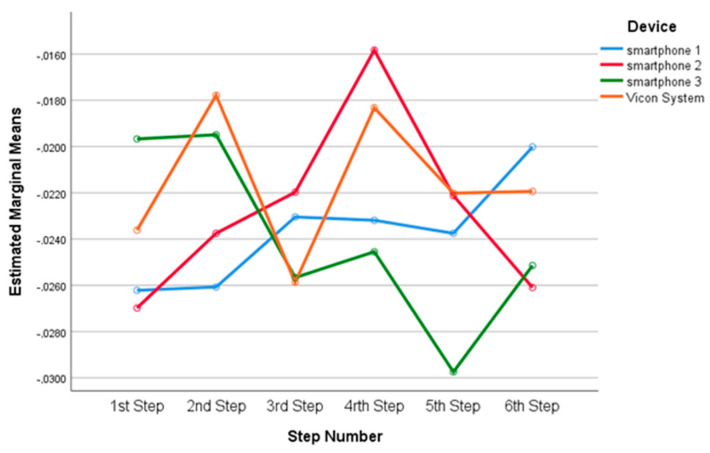
Estimated marginal means for devices among the six steps.

There was no statistically significant two- way interaction between device and step, F_(15,144)_ = 1.162, *p* = 0.308 (Figure 11).

There was no statistically significant two-way interaction between axis and step, F_(10,96)_ = 1.210, *p* = 0.294. There was, also, no statistically significant simple main effect of the factor device F_(3,144)_ = 0.704, *p* = 0.551.

## 4. Discussion

This study sought to identify whether accelerator accuracy obtained with three new state-of-the-art smartphone accelerometers was or was not statistically different from that of the gold-standard Vicon MX motion capture system. We found that the tested smartphone accelerometers seem to be valid and reliable devices for estimating linear accelerations. The terms’ reliability and validity describe psychometric properties of a measuring instrument. Although they are closely related concepts, they express different properties of the measuring instrument. A valid measuring instrument is one that measures the behavior or quality it is intended to measure. A reliable measuring instrument is one that is stable and consistent over time. Consequently, a valid and reliable measuring instrument ensures the quality of measurement and data collected.

We then predicted that the accuracy of the three modern smartphone accelerometers do not differ from that of the Vicon MX motion capture system. Results showed that there were not significant differences between the three current generation smartphone and the Vicon MX motion capture system mean acceleration data.

This evidence extends and advances previous research endeavors on performance evaluation of different commercially available smartphone accelerometers [13,19,20,21,22,23,24,25,26,27,28,29] and indicates how well recent generation smartphone accelerometer sensors are capable of measuring human body motion, which although is not exceptionally rapid, is decidedly complex. Our findings further suggest that off-the-shelf modern smartphone accelerometers (a) can be used as valuable and effective devices for measuring movement under various conditions of performance, (b) can be employed as useful tools for experimental and clinical research, (c) can open up new possibilities for sensing research, (d) can improve and optimize testing methods, and (e) can substitute or take the role of sophisticate scientific instruments when certain circumstances are met (e.g., unavailability of expertise, facilities, money and time).

It should be indicated here, however, that smartphone accelerometer sensors were initially developed to meet the needs of the consumer electronics market—not as scientific sensing systems for measuring human body motion, but as self-sensing devices aware of their surrounding environment in real time for common smartphone functions (e.g., for changing the device’s screen orientation vertically or horizontally). Following the evolution of the smartphone, from being “phone-centric” to being “data-centric” [2], smartphone accelerometer sensors have demonstrated extreme capabilities in quantifying human motor behavior, with potentially far-reaching applications in research and clinical areas [39].

This study, therefore, bridges a significant information gap between the accuracy of accelerometers measured close to production and their accuracy in actual smartphone research. After all, for accelerometer data to be useful to researchers, it must be accurate, valid and reliable indicator of behavior. A large amount of error in the data from the implemented devices will inevitably make conclusions drawn from them unreliable [29].

In general, the current study can serve as a foundation for creating basic technical standards for smartphone accelerometry in human subjects. Additionally, it can be employed to emphasize the state of commercially available smartphone accelerometry technology in terms of technical requirements and capture environment needs that might act as obstacles to the experimental and clinical adoption of human body motion capture. Furthermore, it can guide improvements in the structure and functionality of smartphone accelerometry technology in order to enable broader adoption of these devices in experimental and clinical fields. Finally, it can suggest testing methods of measuring human motor behavior with high ecological validity in naturalistic settings in which the behavior being measured would normally operate. Whether these expectations are realistic or not, presently remains an open question that will require further experimental and clinical experimentation.

Albeit not the focus of this study, it should be briefly mentioned, however, that as with any complex technological system, motion capture systems are not without strengths and weaknesses in theory and in application. Each one has its distinctive technological merits and limitations and, therefore, it is not entirely correct to consider that one system surpasses the other overall, or each system stands on its own as an amazing piece of technological innovation. Consequently, the motion capture system that is chosen for a particular investigation depends on the researcher’s objectives, theoretical background, methodological expertise, time, prior knowledge of the phenomenon being studied, available resources and decision to study a phenomenon connected or separated from its context (e.g., in a natural or a controlled environment). The strengths and weaknesses of the motion capture systems used in the current study, presented below in Table 7, can be applied to inform implementation of experimental and clinical motion capture research and development.

This research was limited by the number of smartphone accelerometers tested. Although selected for comparison with the situation in typical smartphone studies, the study design did not cover the full range of possible devices. However, this was not the purpose of this study. Another limitation was the nature of the design. The study employed single-subject design and, as with all single-subject design studies the external validity of these results requires verification through systematic replication before they can be safely applied as generalizations. Furthermore, whenever a single-subject design is used, the small sample size restricts the type of data analyses that can be undertaken. Nonetheless, this type of design accords with the state of art of gait analysis kinematics variability assessment studies (e.g., [40,41]). Notwithstanding these limitations, it is expected that this study will contribute to the evidence base around the accuracy of smartphone accelerometers and will open new research avenues for future exploration.

Future studies could aim to expand the number of devices tested, as well as to enlarge the sample size investigated to enhance the external validity of the research and to allow more robust statistical analysis of the data. Increasing the type of investigated user devices beyond smartphones and to other, popular or emerging mobile and wearable devices, such as tablets, smartwatches, smart wristbands, smart headbands and smart earbuds, would be a natural extension of the study. Nonetheless, additional research is warranted to validate our arguments, suggestions, and results, since this is the first study on this topic.

Overall, smartphone sensor technology is a highly dynamic and fast-evolving field, limited only by scientific imagination which means, in essence, that anything developed now will be outdated by the time it is available. To paraphrase Miller [2], “the question is not whether smartphone accelerometer sensors will revolutionize motion capture research, but how, when, and where the revolution will happen.

## 5. Conclusions

This study investigated the hypothesis that accelerator accuracy obtained with three current generation smartphone accelerometers and the gold-standard Vicon MX motion capture system was not statistically different. Results demonstrated two findings. First, the tested smartphone accelerometers are valid and reliable devices for estimating accelerations. Second, there were not significant differences among the three current generation smartphone and the Vicon MX motion capture system mean acceleration data. The evidence presented here indicates how effectively modern smartphone accelerometer sensors can measure and quantify human body motion, despite the fact that these sensors are designed not for gathering motor behavior data, but for common smartphone functions. Our work brings new insights to the understanding of the smartphone sensor validity, reliability and accuracy and suggests interesting perspectives for experimental and clinical research. However, much is still unknown and requires further investigation. Nonetheless, studies on this topic can help to inform experimental and clinical scientist how to better achieve desired performance outcomes.

## Figures and Tables

**Table 1:** Smartphone accelerometer properties.

Property	Smartphone 1	Smartphone 2	Smartphone 3
Sensor maker	Bosch Sensortec	STMicroelectronics	STMicroelectronics
Sensor Model	BMI260	LSM6DSL	LSM6DSM
Phone Maker, model	iPhone 12 Pro Max, 5G, IOS 14	Samsung Galaxy S21 Ultra, 5G, Android 11	Huawei P Smart, 5G, Android 10
Type	MEMS	MEMS	MEMS
Sensitivity error	±0.4%	±0.4%	±0.4%
Acceleration Range	±2/±4/±8/±16 g	±2/±4/±8/±16 g	±2/±4/±8/±16 g
Angular Range	±125/±245/±500/±1000/±2000 dps	±125/±245/±500/±1000/±2000 dps	±125/±250/±500/±1000/±2000 dps
Linear acceleration zero-g level offset accuracy	±20 mg	±40 mg	±40 mg
Linear acceleration self-test output change	N/A	90–1700 mg	90–1700 mg
Linear acceleration output data rate	12.5 Hz … 1.6 kHz	1.6 … 6664 Hz	1.6 … 6664 Hz
Rate noise density in high performance mode	160 µg/√Hz 0.008 dps/√Hz	4 mdps/√Hz	3.8 mdps/√Hz
Acceleration g for 0.2 ms	10,000 g	10,000 g	10,000 g
Analog supply voltage	1.71 V to 3.6 V	1.71 V to 3.6 V	1.71 V to 3.6 V

**Table 2:** Acceleration data for the three axis during the six steps trials.

			Device			
Mean Acceleration	No of Steps	Trials	Smartphone 1 (Mean ± SD)	Smartphone 2 (Mean ± SD)	Smartphone 3 (Mean ± SD)	Vicon System (Mean ± SD)
X axis	1	9	−0.0497 ± 0.0050	−0.0492 ± 0.0048	−0.0487 ± 0.0041	−0.0495 ± 0.0042
	2		−0.0512 ± 0.0016	−0.0487 ± 0.0035	−0.0466 ± 0.0037	−0.0468 ± 0.0043
	3		−0.0484 ± 0.0035	−0.0506 ± 0.0037	−0.0476 ± 0.0046	−0.0496 ± 0.0029
	4		−0.0486 ± 0.0040	−0.0493 ± 0.0037	−0.0496 ± 0.0050	−0.0495 ± 0.0030
	5		−0.0488 ± 0.0047	−0.0486 ± 0.0036	−0.0507 ± 0.0038	−0.0493 ± 0.0049
	6		−0.0495 ± 0.0033	−0.0499 ± 0.0043	−0.0518 ± 0.0020	−0.0490 ± 0.0053
	Total	54	−0.0494 ± 0.0038	−0.0494 ± 0.0038	−0.0492 ± 0.0042	−0.0489 ± 0.0041
Y axis	1	9	−0.0237 ± 0.0083	−0.0286 ± 0.0091	−0.0279 ± 0.0099	−0.0287 ± 0.0060
	2		−0.0287 ± 0.0088	−0.0314 ± 0.0074	−0.0305 ± 0.0080	−0.0241 ± 0.0072
	3		−0.0274 ± 0.0076	−0.0309 ± 0.0089	−0.0279 ± 0.0064	−0.0226 ± 0.0072
	4		−0.0282 ± 0.0110	−0.0269 ± 0.0073	−0.0292 ± 0.0069	−0.0220 ± 0.0077
	5		−0.0294 ± 0.0070	−0.0246 ± 0.0062	−0.0272 ± 0.0079	−0.0291 ± 0.0083
	6		−0.0270 ± 0.0090	−0.0269 ± 0.0094	−0.0231 ± 0.0066	−0.0236 ± 0.0051
	Total	54	−0.0274 ± 0.0085	−0.0282 ± 0.0081	−0.0276 ± 0.0077	−0.0250 ± 0.0073
Z axis	1	9	−0.0053 ± 0.0259	−0.0031 ± 0.0245	0.0176 ± 0.0274	0.0074 ± 0.0311
	2		0.0017 ± 0.0351	0.0088 ± 0.0291	0.0186 ± 0.0199	0.0176 ± 0.0166
	3		0.0066 ± 0.0249	0.0155 ± 0.0309	−0.0014 ± 0.0197	−0.0054 ± 0.0312
	4		0.0073 ± 0.0268	0.0287 ± 0.0203	0.0051 ± 0.0299	0.0165 ± 0.0309
	5		0.0070 ± 0.0270	0.0067 ± 0.0246	−0.0113 ± 0.0115	0.0124 ± 0.0240
	6		0.0164 ± 0.0283	−0.0015 ± 0.0251	−0.0005 ± 0.0253	0.0068 ± 0.0281
	Total	54	0.0056 ± 0.0276	0.0092 ± 0.0270	0.0047 ± 0.0245	0.0092 ± 0.0273

**Table 3:** Pearson r correlations from each device linear acceleration’s data on the X axis.

		X Axis Smartphone 1	X Axis Smartphone 2	X Axis Smartphone 3	X Axis Vicon
X axis smartphone 1	Pearson Correlation	1	−0.386	−0.206	0.409
	Sig. (2-tailed)		0.001	0.084	0.039
	N	71	71	71	71
X axis smartphone 2	Pearson Correlation	−0.386	1	0.460	−0.266
	Sig. (2-tailed)	0.001		0.000	0.025
	N	71	71	71	71
X axis smartphone 3	Pearson Correlation	−0.206	0.460	1	−0.464
	Sig. (2-tailed)	0.084	0.000		0.000
	N	71	71	71	71
X axis Vicon	Pearson Correlation	−0.409	−0.266	−0.464	1
	Sig. (2-tailed)	0.039	0.025	0.000	
	N	71	71	71	71

**Table 4:** Pearson r correlations from each device linear acceleration’s data on the Y axis.

		Y Axis Smartphone 1	Y Axis Smartphone 2	Y Axis Smartphone 3	Y Axis Vicon
Y axis smartphone 1	Pearson Correlation	1	0.212	−0.163	−0.415
	Sig. (2-tailed)		0.075	0.175	0.000
	N	71	71	71	71
Y axis smartphone 2	Pearson Correlation	0.212	1	0.239	−0.354
	Sig. (2-tailed)	0.075		0.045	0.002
	N	71	71	71	71
Y axis smartphone 3	Pearson Correlation	−0.163	0.239	1	0.292
	Sig. (2-tailed)	0.175	0.045		0.001
	N	71	71	71	71
Y axis Vicon	Pearson Correlation	−0.415	−0.354	0.392	1
	Sig. (2-tailed)	0.000	0.002	0.001	
	N	71	71	71	71

**Table 5:** Pearson r correlations from each device linear acceleration’s data on the Z axis.

		Z Axis Smartphone 1	Z Axis Smartphone 2	Z Axis Smartphone 3	Z Axis Vicon
Z axis smartphone 1	Pearson Correlation	1	0.304	−0.232	−0.306
	Sig. (2-tailed)		0.010	0.052	0.009
	N	71	71	71	71
Z axis smartphone 2	Pearson Correlation	0.304	1	−0.078	−0.255
	Sig. (2-tailed)	0.010		0.518	0.032
	N	71	71	71	71
Z axis smartphone 3	Pearson Correlation	−0.232	−0.078	1	−0.330
	Sig. (2-tailed)	0.052	0.518		0.002
	N	71	71	71	71
Z axis Vicon	Pearson Correlation	−0.306	−0.255	−0.330	1
	Sig. (2-tailed)	0.009	0.032	0.002	
	N	71	71	71	71

**Table 6:** Intraclass Correlation Coefficient values among device’s acceleration magnitude.

		AM Smartphone 1	AM Smartphone 2	AM Smartphone 3	AM Vicon
AM smartphone 1	ICC	-	0.491	0.632	−0.348
	Sig.	-	0.003	0.977	0.008
	N	-	71	71	71
AM smartphone 2	ICC	0.491	-	−0.110	0.796
	Sig.	0.003	-	0.666	0.001
	N	71	-	71	71
AM smartphone 3	ICC	0.632	−0.110	-	0.270
	Sig.	0.977	0.666	-	0.001
	N	71	71	-	71
AM Vicon	ICC	−0.348	0.796	0.270	-
	Sig.	0.008	0.001	0.001	-
	N	71	71	71	-

**Table 7:** Strengths and weaknesses of the study’s motion capture systems.

System	Strengths	Weaknesses
Vicon MX	Large application segment Unlimited volume data Direct measurement of spatiotemporal variables Multiple performance capture Suitable for extensive motion capture research Long history of use in research	Expensive Time-consuming and complex to operate Small capture area Needs checkerboard calibration procedures Needs controlled environment Low external validity Needs markers on the body Needs line of sight “capture environment Limited movement freedom Unobtrusiveness for users Noise sources and environmental interference
Smartphone Accelerometers	Inexpensive Fast and simple to operate Big capture area Extremely portable and compact Automatic processing No need for a controlled environment High external validity No need for calibration procedures No need for markers on the body No need for line of sight “capture environment” Unlimited movement freedom Unobtrusiveness for particants No noise sources and environmental interference	Small application segment Limited volume data Indirect measurement of spatiotemporal variables Single performance capture Not suitable for extensive motion capture research Brief history of use in research

## Data Availability

The datasets generated during and/or analyzed during this study are not openly available due to reasons of sensitivity (e.g., human data) and are available from the corresponding author upon reasonable request.
